# Impalement injury of the perineum with an iron rod with a minimal injury: A near miss: A case report

**DOI:** 10.1016/j.ijscr.2021.105645

**Published:** 2021-02-11

**Authors:** Bibek Man Shrestha, Dinesh Prasad Koirala, Suraj Shrestha, Sanjeev Kharel, Shankar Raj Lamichane, Geha Raj Dahal

**Affiliations:** a Maharajgunj Medical Campus, Institute of Medicine, Kathmandu, Nepal; b Department of Pediatric Surgery, Tribhuvan University Teaching Hospital, Kathmandu, Nepal

**Keywords:** Emergency, Impalement, Perineum, Trauma, Case report

## Abstract

**Introduction and importance:**

Penetrating perineal injury in children is uncommon. However, the injury is serious and life-threatening with significant morbidity and mortality.

**Case presentation:**

We report an unusual case of a 13-year-old boy with an accidental perineal impalement injury by an iron rod, which pierced through the perineum and exited through the left loin, however with minimal external and internal injuries.

**Clinical discussion:**

Multiple vital organs in the pelvic space are vulnerable to damage in perineal impalement injury. Vital organ injury, amount of blood loss, and effectiveness of the resuscitation determine the outcome of an impalement injury. Urgent laparotomy forms the cornerstone in management as all the potential injuries can be identified and managed immediately.

**Conclusion:**

Effective resuscitation and accurate assessment of the associated injuries along with proper pre-hospital care with a multidisciplinary approach is crucial for the survival and optimum outcome of the victim.

## Highlights

• Perineal impalement injuries are often fatal.
• We report a perineal impalement injury by an iron rod piercing the perineum with minimal external and internal injuries.
• Urgent laparotomy to manage all the potential injuries along with effective resuscitation and prehospital care are critical.

## 1 Introduction

Penetrating perineum injuries are rare emergencies in children with the reported incidence of 4–6 % of injuries of any etiology [1,2]. Impalement injury constitutes the penetration of the body by an object such as a rod, pole, etc, alongside the complete perforation of a central body mass. These types of injuries are often life-threatening with high morbidity and mortality owing to their complex injury patterns and risk of massive pelvic bleeding [3]. Herein, we report a case of a 13-year-old boy who sustained a minimum injury despite penetrating construction iron rod injury traversing from the perineum to the left loin. The case has been reported in line with the SCARE criteria [4].

## 2 Case presentation

A thirteen-year-old boy presented to the emergency department with pain and bleeding from the perineal region along with the penetrating construction iron rod in situ in the perineal region for 15 h after sustaining a fall injury from the rooftop. He was stuck at the top of the iron bar after injury and his relatives cut the bar from its base supporting him from the adjoining house to prevent any possible movements and rushed him to the nearby hospital, and was referred to our center for further management.

**Fig. 1:**
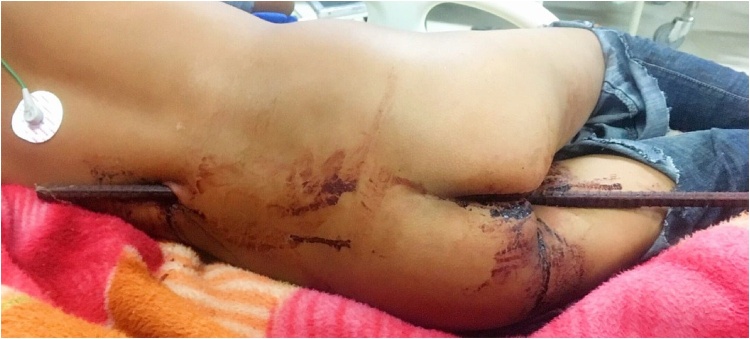
Iron rod penetrating the perineum with an exit site from the left loin.

On arrival to our Emergency Department, the patient was conscious, tachycardic (HR-140bpm), tachypneic, blood pressure of 120/80 mmHg, and other vitals stable. The patient did not any have relevant past history. However, he was bleeding from the perianal region, and a broken metal rod of about 2 cm diameter in situ with entry and exit wound in perineal and left loin region respectively was present (Fig. 1). Emergency management was done according to advanced trauma life support (ATLS) guidelines. Urinary catheterization and rectal examination couldn’t be performed due to the injury and presence of the rod. On local examination, the rod of 2cm × 2cm thickness entered the perineal region 2 cm above the anal opening along with an exit wound of 2cm × 2cm in the left loin region (Fig. 1). Tetanus vaccination and broad-spectrum antibiotics with aerobic and anaerobic coverage were administered.

**Fig. 2:**
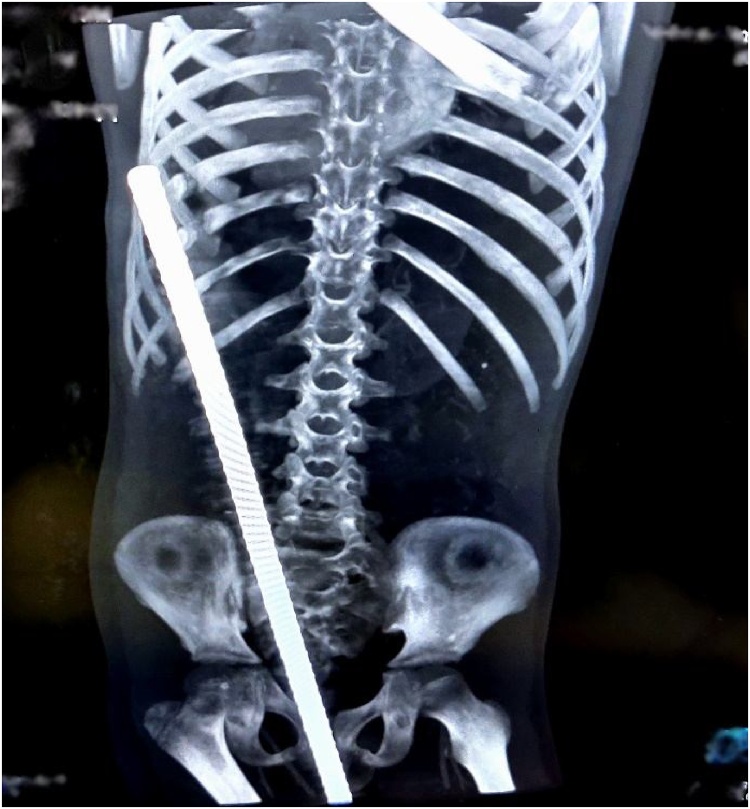
CT scan (coronal view) showing metallic foreign body (rod) extending from right side of perineum to left upper abdomen.

**Fig. 3:**
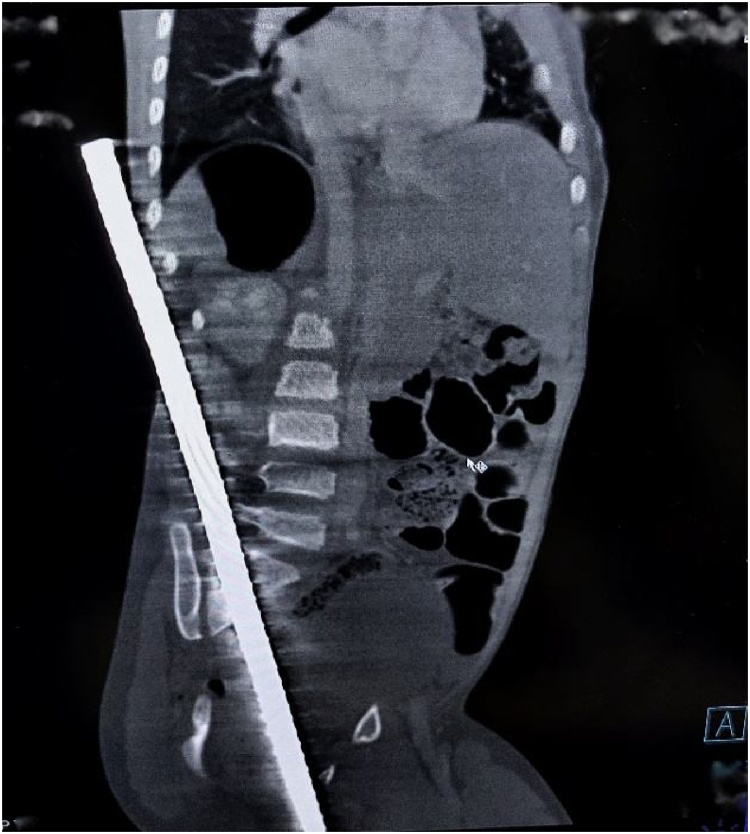
CT scan (oblique sagittal section) showing high density foreign body (rod) passing adjacent to S1 vertebrae from perineum to loin.

Considering hemodynamic stability, radiographic investigations were performed. X-ray of the cervical and dorsal spine revealed no spinal injury and chest X-ray was normal. Computed Tomography (CT) scan of the abdomen and pelvis revealed a metallic foreign body (iron rod) of the approximate diameter of 18 mm penetrating through the perineal region and coursing posterosuperior to the left, posteriorly to the urinary bladder, anterior to left ala of sacrum, left anterolateral aspect of the rectum, close to the left transverse process of L_4_-L_5_ vertebrae, along the left paraspinal region and then exiting outside of the left posterior upper abdominal wall at T10 vertebrae level with no obvious injury of the urinary bladder, rectum and other visceral organs and major large arteries (Fig. 2, Fig. 3).

**Fig. 4:**
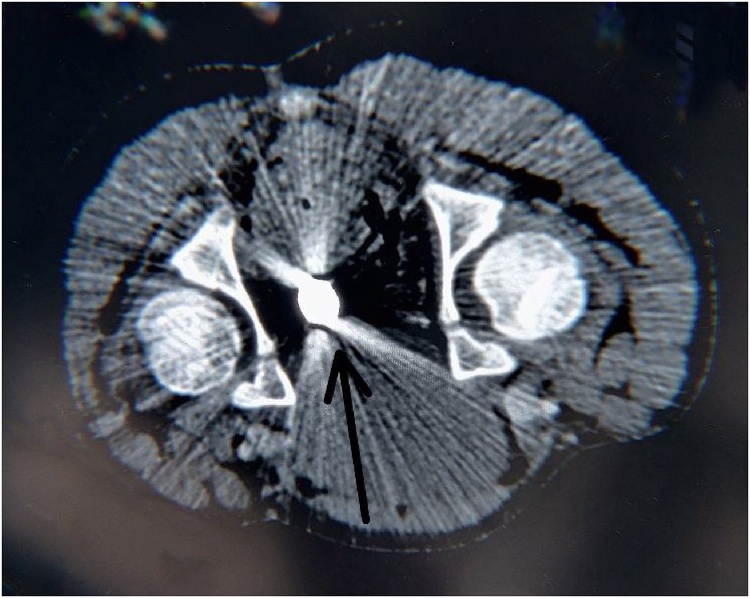
CT scan (transverse section) showing foreign body (rod) posterior to Urinary bladder and anterior to rectum.

**Fig. 5:**
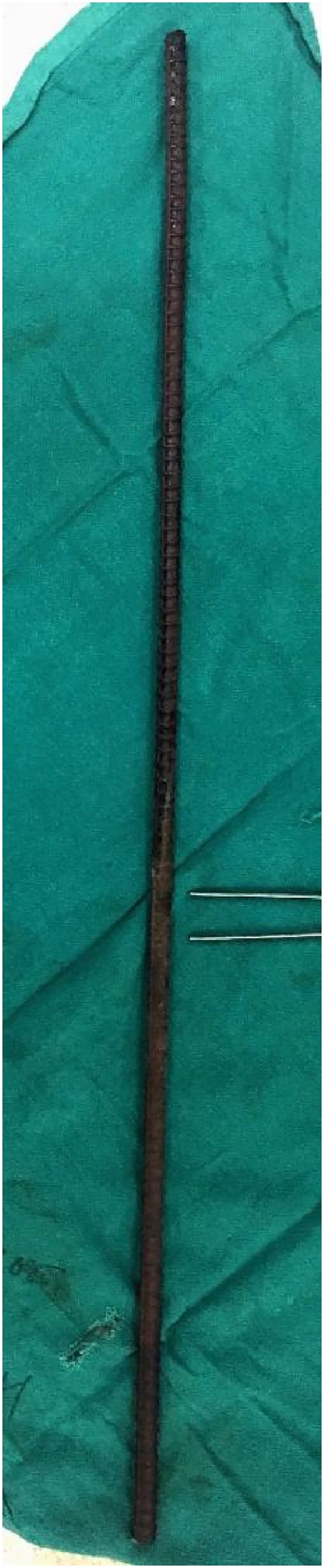
Iron rod removed from the body after the surgery.

Considering the possibility of rectosigmoid/retroperitoneal injury, an exploratory laparotomy was done with lower midline access and after meticulous observation, no injuries to vital organs by the rod were found. Perioperatively, 2 cm × 2 cm thickness iron rod was passing through the perineal body with penetration of urogenital diagram passing through the iliopsoas muscle till the lower border of the kidney with exit wound of 2 cm × 2 cm in the left lumbar region (Fig. 4). No pathological findings other than about 100 ml of clotted blood in the peritoneal cavity along with a small perforation in the transverse colon was detected. Surprisingly, there was no injury to the rectum, urinary bladder, small bowel, kidney, spleen, major vessels, urethra, external genitalia, and external anal sphincter. The rod was removed slowly and steadily under direct vision ensuring no more damage (Fig. 5). The perforation of the transverse colon and the defect in the urogenital diaphragm were repaired. The perineal wound was cleaned and allowed to heal secondarily and a long Penrose drain was placed into the entry hole. The lumbar exit hole was closed. The procedure was performed by experienced pediatric surgeons’ team of Tribhuvan University, Teaching Hospital encountering no any other complications and was well tolerable by the patient.

The patient recovered well and was discharged on the 9th postoperative day, having remained in the pediatric intensive care unit for four days, without any complications related to walking, urination, and defecation. On follow up after 3 weeks, wounds were healthy and the perineal wound had healed.

## 3 Discussion

Perineal impalement injury is catastrophic. As in this case, falling on the offending objects are often responsible for the injury. Other etiologies include physical assaults and sexual abuse [5,6]. As high as 25 % of victims die before reaching the health facility or within a few hours after the incident due to uncontrollable hemorrhage [7]. Also, those who survive massive resuscitation, and even after reaching hemostasis, are prone to develop septic complications, which can appear in up to 80 % of patients with perineal trauma [8]. Thus, the presence of vital organ injury, amount of blood loss, and adequacy of the resuscitation along with control of the sepsis determine the prognosis of the injury [5]. In addition it appears that the pediatric population has a more favorable outcome than the adult population with respect to these injuries [9].

Pre-hospital care is critical for the survival of these patients. Since the impaling object may have a tamponade effect on the organs, preventing the bleeding, through which it has penetrated, the impaling object must only be removed under direct vision in a controlled environment such as in the operating room [10]. Shortening of the object may however be attempted to facilitate transport. Also, the impaling object must be secured to prevent any movement in relation to the body of the patient such that further soft-tissue damage and bleeding can be prevented [6]. Similarly, in our case, the iron rod was cut with minimal movement to facilitate transport and no attempt was made to remove it until the patient was shifted to the operation room.

The location of the entry wound, angulation, and intensity are the factors that affect the trajectory of the object and the involvement/injuries of adjacent organs [11]. This might probably affect the depth and impact it can have on the inner body structures as the traumas are not only limited to the anorectal region but can affect every region that the foreign body passes through. This mandates a thorough examination.

Considering the complexity of the injury and the rarity of the case, there are no established standard treatment guidelines. Every trauma patient arriving at the Emergency department should initially be treated in accordance with the ATLS protocol and so was done in our case.

CT scan is considered the first-line investigation in perineal trauma and CT abdomen and pelvis should be done in all patients which allows the internal injuries to be identified quickly and prioritized for appropriate management [12]. Additional appropriate investigations can be done in hemodynamically stable patients with normal contrast-enhanced CT (CECT) findings [13]. In our case, both CXR and CECT were performed.

The anatomically restricted pelvic space and presence of numerous vital anatomic structures make this region highly vulnerable for acquiring an internal organ injury, with studies reporting up to 85 % chance [14]. Because the impaling objects usually traverse multiple body cavities and can predispose to multiple organ injuries, most of the cases have to be treated operatively, however, a few cases have been managed conservatively [14]. As severe problems are expected with the injury of intra-abdominal organs, early surgery/intervention is critical for improving sepsis and wound recovery, particularly within 6 h [15]. In our patient, the time to surgery was approximately 20 h.

Urgent laparotomy is the safest and the most conservative approach because it helps to identify and manage all potential injuries immediately [16]. Because the entry site does not necessarily determine the extent and type of organ injuries, exploration of the involved cavities is often necessary [17]. Additionally, impalement injury causes soft tissue necrosis, thorough debridement of necrotic tissue and irrigation of the site of the wound and cavities the object has traversed is paramount [10]. Also, to reduce the high risk of infection in a contaminated wound, the wound should not be closed primarily [17]. To minimize and avoid any mortality, in every case of penetrating perineal injury, a combined rectal and urinary tract injury should be ruled out [18]. The iron rod in our case was removed in a controlled way, the wound site and the rod track were irrigated and the entry site was allowed to heal secondarily.

Meticulous presacral drainage, distal rectal wash, appropriate rectovesical wound repair can decrease injuries related complications. Moreover, prolonged suprapubic drainage and isolating rectal and urinary tract wounds can help prevent complications [19]. Along with the sphincter, urinary system problems, and vital organ injuries, perineal injuries also require long-term follow-up [20]. Our patient is on close follow-up, prospering and satisfied with treatment.

## 4 Conclusions

Perineal penetrating injuries are uncommon with high morbidity and mortality. Early effective resuscitation and accurate assessment of the associated injuries along with proper pre-hospital care are decisive in improving patients' survival. A multidisciplinary approach is required involving different surgical specialties for the optimal outcome.

## Declaration of Competing Interest

None

## Funding

None

## Ethical approval

Nothing to declare.

## Consent

Written informed consent was obtained from the patients’ father for publication of this case report and accompanying images. A copy of the written consent is available for review by the Editor-in-Chief of this journal on request

## Authors contribution

Bibek Man Shrestha (BMS), Dinesh Koirala (DK), and Gehraj Dahal (GD)= Study concept, Data collection, and surgical therapy for the patient

BMS and Suraj Shrestha (SS) =Writing- original draft preparation

Sanjeev Kharel (SK) and Sankar Raj Lamichane (SRL)= Editing and writing

DK and GD = Senior author and manuscript reviewer

All the authors read and approved the final manuscript.

## Registration of research studies

Not Applicable

## Guarantor

Bibek Man Shrestha

## Provenance and peer review

Not commissioned, externally peer-reviewed.
